# Pathogenesis and Transmissibility of Middle East Respiratory Syndrome Coronaviruses of African Origin in Alpacas

**DOI:** 10.3390/v17111524

**Published:** 2025-11-20

**Authors:** Richard A. Bowen, Airn Hartwig, Anneliese Bruening, Audrey Walker, Malik Peiris

**Affiliations:** 1 Department of Biomedical Sciences, Colorado State University, Fort Collins, CO 80523, USA; 2 School of Public Health, The University of Hong Kong, Hong Kong SAR, China

**Keywords:** MERS-CoV, Middle East Respiratory Syndrome, transmission, coronavirus, alpaca

## Abstract

The Middle East Respiratory Syndrome coronavirus (MERS-CoV) remains a highly significant threat to global public health. Dromedary camels are the zoonotic source of human infection. All cases of zoonotic Middle East Respiratory Syndrome (MERS) have occurred in Middle Eastern countries despite MERS-CoV infection of camels being widespread in Africa. This disparity in the geographic burden of the disease may be due to genomic differences between MERS-CoV circulating in Middle Eastern countries (clades A and B) versus those infecting camels in Africa (clade C), although the precise genetic determinants of virulence remain to be elucidated. The objective of the studies reported here was to evaluate differences in the magnitude of virus shedding and in transmissibility of clades A/B and C viruses using alpacas as a surrogate for dromedary camels. We found that two of three African-origin, clade C strains of MERS-CoV induced very reduced levels of virus shedding and were transmitted inefficiently to contact control animals as compared to one other clade C virus and representative viruses from clade A and B. Lower virus titers in the nasopharynx may be associated with lower zoonotic transmission and human disease severity and may explain the observed epidemiology of MERS-CoV in Africa where zoonotic disease appears rare. These results add to our understanding of the transmission of different lineages of MERS CoV in camelids and zoonotic transmission.

## 1. Introduction

The Middle East Respiratory Syndrome (MERS) results from infection with a betacoronavirus (MERS-CoV) that was first reported in 2012 following its isolation from a 60-year-old Saudi man who died of severe respiratory disease [1]. Since then, more than 2600 laboratory-confirmed cases of MERS have been reported to the World Health Organization [2]. A large majority of these cases originated from the Arabian Peninsula, although cases have been reported from at least 27 other countries, most frequently associated with travelers outside the Middle East [2]. MERS is a zoonotic disease, and MERS-CoV may have evolved from a bat virus [3], but the critical epidemiologic feature of the disease is that dromedary camels serve as the source of zoonotic disease and amplifying host for the virus [4,5,6,7,8,9]. Infected camels show no or very mild clinical signs of disease, but can shed high quantities of virus in nasal secretions, and contact with infected camels appears to be the major source of infection for humans. Serologic testing of archived camel sera makes it clear that MERS-CoV infection of dromedaries has occurred in the Middle East and Africa since at least 1992 and likely earlier [10,11].

A large majority of the world’s dromedary population resides in Africa, and several studies have documented the presence of MERS-CoV in dromedaries from multiple African countries either by detection of virus or viral RNA, or through serologic testing [10,11,12,13,14,15,16,17,18,19]. Interestingly, zoonotic MERS disease in humans in Africa has not been reported, although antibodies to the virus in humans, especially those having close contact with camels, have been reported [19,20,21,22]. The disparity in the incidence of human disease between the Middle East and Africa may be due to virus genotype. Currently, three clades of MERS-CoV have been defined [15]. The original 2012 isolate of MERS-CoV is a representative of clade A, which apparently no longer circulates. Clade B viruses are dominant in camels and humans from Middle Eastern countries, while clade C viruses are found exclusively in Africa. The replication capacity of several representatives of these clades has been compared in cultured human respiratory cells (Calu-3), ex vivo cultures of human respiratory tract and the lungs of human dipeptidyl peptidase-4 (DPP4) transgenic mice, and the clade C viruses replicated to a significantly lower extent than clade A and B viruses in these experimental systems [23,24].

A comparison of virus replication competence, virus shedding, and transmission of different virus lineages in the camelid host would enhance our understanding of the epizootiology and zoonotic potential of these viruses. Experimental infection of camels with MERS-CoV has been performed by multiple groups [25,26,27], but it is expensive, dangerous to personnel, requires specialized BSL3 facilities, and only a limited number of viruses can be compared. Previously, we established an alpaca model for MERS-CoV infection that is considered a viable alternative for evaluating pathogenesis and transmission and is considerably more amenable to experimental studies than the use of camels [28]. This alpaca model was exploited for the current studies to compare the pathogenesis of infection and transmissibility of clade A and B viruses with three African-origin clade C viruses.

## 2. Materials and Methods

### 2.1. Animals

A total of 23 alpacas, aged 3 to 8 years of age, were purchased from a private source in Colorado, USA, and assigned non-randomly to treatment groups to minimize disparity in age among groups. The animals were housed in a large animal ABSL3 facility for at least one week prior to inoculation with MERS-CoV and fed a diet of alfalfa hay supplemented with mixed grain. During the acclimatization period, each alpaca was implanted subcutaneously with a thermally sensitive microchip (LifeChip, Destron-Fearing, DFW Airport, TX, USA) for identification and monitoring body temperature.

### 2.2. Viruses

The four viruses were provided by co-author MP, and their genetic sequences have been described previously [23,24]. Each of the virus stocks received was passaged one additional time in Vero E6 cells to generate the working stocks that were used for animal inoculation. Virus characteristics are described in Table 1.

### 2.3. Virus Titration and Antibody Assays

Procedures used for titrating the virus and determining neutralizing antibodies were the same as described previously [28]. The virus was titrated to confirm the inoculation dose and to determine the magnitude of virus shedding based on nasal swab samples. In all cases, a standard double overlay plaque assay on Vero E6 cells was used. Samples were serially 10-fold diluted in BA1 medium (Tris-buffered minimal essential medium supplemented with 1% bovine serum albumin) containing 5% fetal bovine serum (FBS), 50 μg/mL gentamicin, 100 units/mL penicillin G, 25 μg/mL streptomycin and 2.5 μg/mL amphotericin B. Confluent monolayers of Vero E6 cells grown in 6 well plates were inoculated with 0.1 mL/well of the virus dilutions, incubated for 45 min and overlaid with 2 mL of phenol red-free minimal essential medium containing glutamine, 2.5% fetal bovine, antibiotics as above, and 0.5% agarose. Two days after inoculation, the plates received another identical 2 mL overlay containing 0.005% neutral red. Plaques were counted 1 and 2 days after the second overlay. Virus titers were expressed as plaque-forming units (PFU) per ml. The limit of detection (LOD) for nasal swab virus titrations was 10 PFU/swab.

Neutralizing antibody titers were determined by plaque reduction neutralization test, using 80% reduction as the threshold for positivity (PRNT80). Sera were heat-inactivated for 30 min at 56C and serial two-fold dilutions beginning at 1:5 in BA1 medium were mixed with an equal volume of BA1 containing approximately 180 PFU of the EMC strain of MERS-CoV. After incubation for 60 min, 0.1 mL volumes of the virus-serum mixtures were inoculated onto confluent monolayers of Vero E6 cells as described above for plaque assay.

### 2.4. Study 1

Four groups of two alpacas were infected with four isolates of MERS-CoV: BF 785, Nig 1657, Mor 213, and SA AH13 by instillation into each animal a total volume of 1 mL containing a target dose of 5 log_10_ PFU, for a total dose of 5.3 log_10_ PFU; the pairs of alpacas inoculated with each virus were housed in separate rooms. The alpacas were lightly sedated with xylazine 5–10 min prior to inoculation and their heads elevated vertically during and for approximately 30 s following inoculation. The day of virus inoculation was designated as day 0. Two days following MERS-CoV inoculation, two naïve alpacas were introduced into each room with the two virus-inoculated animals as contact controls to assess transmission. Nasal swabs were collected from the inoculated alpacas on days 1 to 5 and from contact animals on days 3 to 7. These samples were collected by insertion of a polyester-tipped swab approximately 7–8 cm into each nasal cavity, rotating several times, and breaking the tip off into 1 mL of BA1 medium containing 5% FBS; these samples were frozen to −80 °C until they were assayed for infectious virus. Each of the swabs was assayed independently without pooling. Blood was collected into evacuated tubes on days −2 and 28, and sera were stored for antibody assay. Body temperature was recorded once daily beginning immediately prior to inoculation and extending through day 7. Animals were humanely euthanized on days 28 or 31.

### 2.5. Study 2

Study 2 was conducted to partially replicate the results from Study 1 with regard to two of the African-origin MERS-CoV and to evaluate a clade B virus. Seven alpacas were available for Study 2. Three pairs of alpacas were infected with each of three MERS-CoV (Mor 213, Nig 1657, and AH13) as described for Study 1. In the case of the two alpacas infected with AH13, an additional naïve alpaca was introduced into their room 2 days after virus inoculation to assess contact transmissibility of that virus. Clinical observations and sampling to obtain nasal swab specimens for virus assays were the same as in Study 1. Serologic responses were not evaluated.

## 3. Results

### 3.1. Study 1

Clinical signs of disease, including overt nasal discharge and even mild fever, were not observed in any of the inoculated or contact control animals during the entire course of the study. Alpacas inoculated with the EMC (clade A) and BF 785 (clade C) viruses shed high quantities of virus nasally starting one day post-challenge and efficiently transmitted infection to the two co-housed contact controls. Nasal virus shedding from the contact controls was first observed 2 to 4 days after their introduction. In contrast, alpacas challenged with the other two clade C viruses (Mor 213 and Nig 1657) showed relatively low virus shedding and, with the exception of one low-titer sample that could have been from contact contamination rather than true virus replication, transmission to contact controls was not observed.

**Figure 1:**
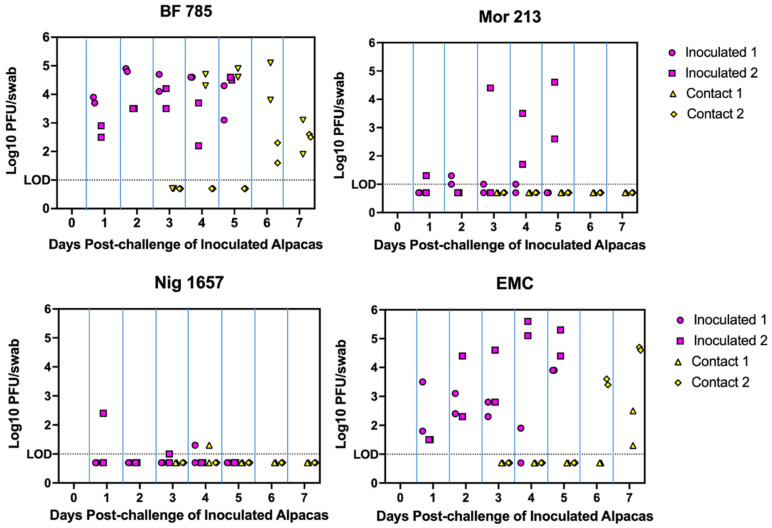
Experiment 1: Nasal virus shedding in alpacas inoculated with each of 4 strains of MERS-CoV (Inoculated) or exposed to virus by co-housing with inoculated animals (Contact). Titers for duplicate swabs collected on each day are shown. Samples were assayed from animals inoculated with MERS-CoV on days 1 to 5 and from contact controls on days 3 to 7.

Virus titers from nasal swabs, representing nasal shedding from both inoculate and contact animals, are depicted in Figure 1.

All the inoculated and contact control alpacas developed a robust neutralizing antibody response by 28 days after virus challenge. This was despite the lack of detectable virus shedding among the contact control animals in the Mor 213 and Nig 1657 rooms, indicating that even though they failed to shed, some virus replication likely occurred and stimulated an immune response. A summary of peak virus shedding, efficiency of transmission, and serologic responses is presented in Table 2.

### 3.2. Study 2

**Figure 2:**
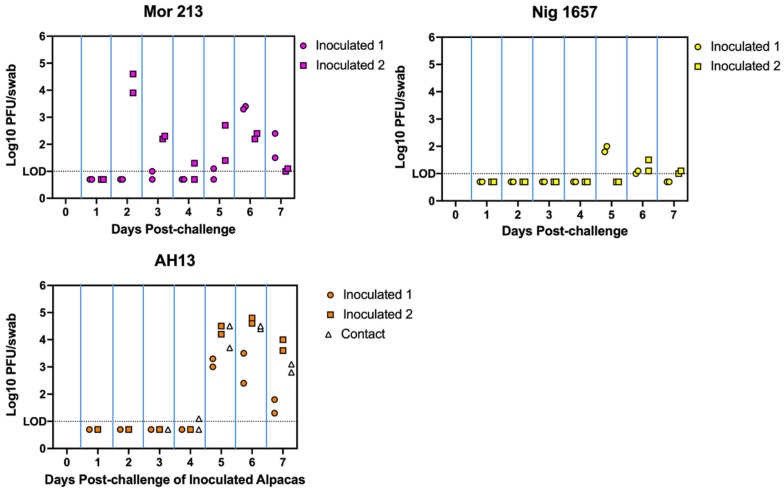
Experiment 2: Nasal virus shedding in alpacas inoculated with each of 3 strains of MERS-CoV (Inoculated) or exposed to virus by co-housing with inoculated animals (Contact, AH13 only). Titers for duplicate swabs collected on each day are shown.

The objective of Study 2 was to replicate infection of alpacas with two of the MERS-CoV strains used in Study 1 (Mor 213 and Nig 1657) and to evaluate the course of infection and transmissibility of a clade B virus strain (AH13). The two animals that were inoculated with Mor 213 and Nig 1657 displayed levels of nasal virus shedding very similar to those found in Study 1. Both alpacas inoculated with the AH13 virus shed high quantities of virus, but not until 5 days after virus inoculation, which was distinctly later than that observed in Study 1 for animals inoculated with BF 785 and EMC viruses. Interestingly, the contact control alpaca that was introduced into the room with the two AH13-inoculated animals began shedding virus at essentially the same time as the two animals directly inoculated with that virus. Virus titers from nasal swabs collected for Study 2 are shown in Figure 2.

## 4. Discussion and Conclusions

MERS-CoV, like its betacoronavirus cousins SARS-CoV and SARS-CoV-2, is among the ten high-priority pathogens designed by the World Health Organization based on their epidemic potential and insufficient countermeasures [29]. Unlike other zoonotic pathogens currently causing global concern (e.g., influenza A H5N1), MERS-CoV has sometimes caused outbreaks in Saudi Arabia and the Republic of Korea within humans with 3–4 cycles of human-to-human transmission affecting over 150 individuals before containment was finally established [30,31]. However, coronaviruses readily mutate by both mutation and recombination [32], and a major concern is that MERS-CoV will evolve to facilitate efficient transmission among humans. Human adaptive mutations associated with zoonotic transmission of clade B MERS-CoV have been reported [33], and diverse genomic deletions and insertions have been observed in MERS-CoV in Africa [34]. Indeed, the distinct differences in zoonotic transmission potential from camels to humans of clade A/B versus C MERS-CoV could be a harbinger of future evolution of this pathogen as a pandemic threat. Similarly, if the clade B MERS-CoV with known zoonotic potential and apparently more efficient transmission between camelids were to be introduced into Africa, it is likely to outcompete existing clade C viruses with major adverse public health consequences. It is therefore important to obtain a more comprehensive understanding of the biologic properties of genetically divergent MERS-CoV.

Replication kinetics and susceptibility of representatives of MERS-CoV from different clades have been reported previously [23,24]. The key observation from these studies is that clade C viruses from West and East Africa were replication-impaired compared to clade A and B MERS-CoV when assessed in cultured human cells, ex vivo explants of human lung, and in the lungs of transgenic mice expressing the human DPP4 virus receptor. There are a substantial number of differences in the genomes of these different variants, and it is not clear which are the critical determinants of replication fitness. Using reverse genetic approaches to address this question, both ORF4b (an interferon antagonist) [23] and the spike protein gene [24] have been proposed to explain the lower replication competence of African clade C viruses compared to clades A and B viruses from Saudi Arabia, but in both studies, the authors suggested that multiple genes are almost certainly involved and a comprehensive understanding of differences in replication competence among clades remains to be established. Importantly, all three clades of MERS-CoV appear antigenically equivalent with regard to neutralization by antibodies, a feature that simplifies serosurveillance in both humans and camels.

In addition to defining how viruses from different clades interact with human cells, it is also important to understand pathogenesis and transmissibility in their reservoir host. As described earlier, it is logistically difficult to do such studies in camels, but alpacas appear to be a valid surrogate for such studies. Experimental infection of alpacas with MERS-CoV and demonstration of virus shedding have been reported previously from multiple groups [28,35,36]. Additionally, serological evidence for susceptibility of alpacas to infection with MERS-CoV in natural settings has been reported for animals from Qatar [37] and Israel [38]. More recently, alpacas were used to show that infection with a clade B MERS-CoV (Jordan-1/2015) resulted in higher levels of shedding, higher burdens of viral RNA in respiratory tissues, and, by immunohistochemistry, a greater density of infected nasal epithelial cells compared to infection with the clade A EMC strain of MERS-CoV [38].

The objective of the current study was to assess differences in virus shedding and transmissibility between three African-origin clade C viruses and representatives from clades A and B using a natural host model for MERS-CoV infection. As expected, we found that infection with clade A (EMC) or clade B (AH13) MERS-CoV resulted in high-level shedding and efficient transmission to contact controls. Infection of alpacas with the BMF 785 clade C virus also led to high-level shedding and transmission to both contact controls. The two other clade C viruses—Mor 213 and Nig 1657—were tested twice for shedding. In both studies, infection Mor 213 virus resulted in moderate nasal shedding, while nasal shedding was minimal following inoculation with Nig 1657 virus. Transmission of both Mor 213 and Nig 1657 to controls introduced days after inoculation was tested in Study 1 and was not observed to occur. When these in vivo results are compared to in vitro studies, the only discordance is seen with BF 785: in alpacas, that virus appeared to replicate and be transmitted similarly to EMC and AH13, but was replication-restricted when tested in human cells, ex vivo human lung, and transgenic mice expressing the human MERS-CoV receptor [23,24]. Two of the three African clade C viruses tested were clearly less competent in vivo in inducing virus shedding and transmission between camelids. Lower MERS-CoV titers in camelid nasopharynx may be associated with lower zoonotic transmission and less human disease severity, and may explain the observed epidemiology of MERS-CoV in Africa, where zoonotic disease appears rare.

A key limitation of the studies presented here was the small number of animals tested and the limited number of viruses evaluated, which precluded valid statistical comparisons. As stated above, the genetic determinants of virulence for clade A/B versus clade C MERS-CoV are poorly defined and likely multifactorial. Considering the large genetic diversity of African strains of MERS-CoV, it would clearly be of value to extend these studies to further delineate differences in pathogenesis using a host relevant to natural zoonotic transmission and a larger battery of MERS-CoV isolates.

## Figures and Tables

**Table 1:** Characteristics of viruses used to inoculate alpacas.

Virus Description	Clade	Passage History *	Abbreviation	Origin
MG923471/camel/Burkina_Faso/CIRAD-HKU785/2015	C	P4	BF 785	Burkina Faso
MG923475/camel/Nigeria/NV1657/2016	C	P5	Nig 1657	Nigeria
MG923469/camel/Morocco/CIRAD-HKU213/2015	C	P7	Mor 213	Morocco
KJ650295/camel/Saudi_Arabia/KFU-HKU13/2013	B	P5	AH13	Saudi Arabia
JX869059/human/EMC/2012	A	P5	EMC	Saudi Arabia

* Total number of cell culture passages for virus stocks inoculated into alpacas.

**Table 2:** Experiment 1: Summary of virus shedding in inoculated and contact control animals, transmission to contacts, and seroconversion. Peak virus shedding is the highest titer detected in swabs collected on days 1 to 5 for inoculated animals and on days 3 to 7 for the contact control animals.

Virus	Exposure Route	Peak Virus Shedding (log10 PFU)	Transmission to Contacts	Day 28 80% Neutralization Titer (PRNT80)	
				Alpaca 1	Alpaca 2
BF 785	Inoculation	4.9	2 of 2	≥320	≥320
BF 785	Contact	5.1		160	≥320
Mor 213	Inoculation	4.6	0 of 2	≥320	160
Mor 213	Contact	Not detected		≥320	160
Nig 1657	Inoculation	2.4	1 (?) of 2	160	80
Nig 1657	Contact	1.3		40	160
EMC	Inoculation	5.6	2 of 2	≥320	160
EMC	Contact	4.7		160	≥320

## Data Availability

The original contributions presented in the study are included in the article; key data have also been included in the article in graphical form. Further inquiries can be directed to the corresponding author.

## Abbreviations

The following abbreviations are used in this manuscript:

DPP4	dipeptidyl peptidase-4
MERS	Middle East Respiratory Syndrome
MERS-CoV	Middle East Respiratory Syndrome coronavirus
PFU	Plaque-forming units
